# Supplementary material for: Curated and harmonised transcriptomics datasets of interstitial lung diseases
Source: Data Brief. 2025 Oct 14;63:112139. doi: 10.1016/j.dib.2025.112139 (PMC12581653; doi:10.1016/j.dib.2025.112139)
Supplement: Supplementary file 1 [file mmc1.zip › Supplementary_material/RNA-seq/fastQC reports/GSE184316/TRIMMED_SRR15926812_fastqc.html]

TRIMMED\_SRR15926812.fastq.gz FastQC Report 

FastQC Report

Sat 3 Dec 2022  
TRIMMED\_SRR15926812.fastq.gz

## Summary

- Basic Statistics
- Per base sequence quality
- Per sequence quality scores
- Per base sequence content
- Per sequence GC content
- Per base N content
- Sequence Length Distribution
- Sequence Duplication Levels
- Overrepresented sequences
- Adapter Content

## Basic Statistics

| Measure | Value |
| --- | --- |
| Filename | TRIMMED\_SRR15926812.fastq.gz |
| File type | Conventional base calls |
| Encoding | Sanger / Illumina 1.9 |
| Total Sequences | 10440979 |
| Sequences flagged as poor quality | 0 |
| Sequence length | 35-372 |
| %GC | 51 |

## Per base sequence quality

## Per sequence quality scores

## Per base sequence content

## Per sequence GC content

## Per base N content

## Sequence Length Distribution

## Sequence Duplication Levels

## Overrepresented sequences

| Sequence | Count | Percentage | Possible Source |
| --- | --- | --- | --- |
| CAGTGGGGCCACGAGCTGAGTGCGTCCTGTCACTCCACTCCCATGTCCCT | 93378 | 0.8943414214318409 | No Hit |
| CACCTCCCCGAAGCTCAGGGAGAGCCCTGTTAGGGCCGCCTCTGGCCCTA | 76457 | 0.7322780746901224 | No Hit |
| AGACATCAAATCCTGCAGACAAGGGGAGCCCTCAGTCTGCAGGGCTCCAT | 67714 | 0.648540716344703 | No Hit |
| GGCATCAAAAGTGATGGACTGCCCATTGCTGGAGAAGACCTTCTCTCCTA | 61097 | 0.5851654332414613 | No Hit |
| AGGCTACACACTGAGGACTCTGTTCCTCCCCTTTCCGCCTAGGGGAAAGT | 56498 | 0.5411178396202119 | No Hit |
| CTTCATGATGTAGCAGCAGGTGCCAGGGGCTGGCTTGTAGGCGATCAGCA | 52157 | 0.4995412786482954 | No Hit |
| TACCACTGCCACCTTCTCCATCGGCTCCACTGGCCTCGTGGTGTATGACT | 49735 | 0.4763442202115338 | No Hit |
| TGATATGGTTCCTGGCAAGCCCATGTGTGTTGAGAGCTTCTCAGACTATC | 42901 | 0.4108905879419928 | No Hit |
| TGATGACACCCACCGCAACTGTCTGTCTCATATCACGAACAGCAAAGCGA | 42186 | 0.404042571103725 | No Hit |
| GGGAAGCGGGGAATGTCTACGTGCGTATGCACGTGGCACTCTCTGCCCGA | 41592 | 0.3983534494227026 | No Hit |
| GTCCACGTCACACTTCATGATGGAGTTGAAGGTAGTTTCGTGGATGCCAC | 32871 | 0.3148267992876913 | No Hit |
| GGCAATGAGCGGTTCCGCTGCCCTGAGGCACTCTTCCAGCCTTCCTTCCT | 32393 | 0.31024868453427595 | No Hit |
| AGCTGCTAAGTGCTGTGTTGTCGTTCCCCCTGCTTAAAATAAAGTTGTTT | 29895 | 0.28632372500701325 | No Hit |
| CCCTGGCTGCTACAGGAGAATAGCAGACAGGTATAGTTAAGAAACAACTT | 29094 | 0.2786520306189678 | No Hit |
| ACCTCTCTCTGGGCTTCTATTTCGACCGCGATGATGTGGCTCTGGAAGGC | 26810 | 0.2567766873202216 | No Hit |
| CTCTCTTTCTGGCCTGGAGGCTATCCAGCGTACTCCAAAGATTCAGGTTT | 24628 | 0.23587826390609543 | No Hit |
| ACAAGAAGTACATCAAAGATTACATGAAATCAATCAAAGGGAAACTTGAA | 23843 | 0.2283598118528923 | No Hit |
| CAGGACCACCAATGTCATAGGGTGCAATATCTACAATAGGTAGTCTCACA | 23692 | 0.22691358731781763 | No Hit |
| CAATGGGGTACTTCAGGGTCAGGATGCCACGCTTGCTCTGGGCCTCGTCG | 23080 | 0.22105206801009752 | No Hit |
| CACCAAAGTGCGAAAAGTTACTTTCAATGGACTGAACCAGATGATTGTCA | 21847 | 0.20924283058130852 | No Hit |
| CGGCGAGAGCATGACCGATGGATTCCAGTTCGAGTATGGCGGCCAGGGCT | 21815 | 0.2089363459116238 | No Hit |
| GGAGGATGGTTGCACGAAACACACTGGGGAATGGAGCAAAACAGTCTTTG | 21769 | 0.20849577419895204 | No Hit |
| GACCGAATTCCCCCTGCGCATGCGGGACTGGCTCAAGAACGTCCTGGTCA | 21417 | 0.20512444283242023 | No Hit |
| CCAAAGGTGCAATATCAAGGAAGGGCAGGCGTGATGGCTTATTTGTTTTG | 21178 | 0.20283538545571253 | No Hit |
| CATATGGCTCTCCCAGAGGCAGAAGGACAATGGCTGTTTCAGGAGCTCTG | 20974 | 0.2008815456864725 | No Hit |
| ACACTGTTCTTGTAGATGGCTGCTCTAAAAAGACAAATGAATGGGGAAAG | 20219 | 0.19365042301109886 | No Hit |
| GAAACCCAGACACATAGCAATTCAGGAAATTTGACTTTCCATTCTCTGCT | 20114 | 0.1926447701886959 | No Hit |
| CTTGCAGAAGCTCTTTATGTCAAACTTCTCAAGTTGATTGACCTCCAGGT | 19874 | 0.19034613516606058 | No Hit |
| CAGCAGTTTACACCTGCTAGGGTGTTCAAAGGTCAGTGCTATAGAAATTC | 19326 | 0.1850975851977099 | No Hit |
| AGGTGATGTTCTGGGAGGCCTCGGTGGACATCAGGCGCAGGAAGGTCAGC | 17903 | 0.17146859504266793 | No Hit |
| ATCAAAGAAGAACATGTGATCATCCAGGCCGAGTTCTATCTGAATCCTGA | 17824 | 0.1707119610143838 | No Hit |
| CGATCAGCACGGGCTCGTACTCTGCCACAAACTGATCACACTGCTTCTGG | 17504 | 0.1676471143175367 | No Hit |
| GTTCTGCAGCCCCTGTCATAAAAGGTTTTACTCTTTCTGGTCTCTGTTCT | 17219 | 0.16491748522815725 | No Hit |
| CAATCCACTTGATCCCAACTCATCTCTCATTTATTTCGGCTTCTTTTATT | 17197 | 0.164706777017749 | No Hit |
| TTACAAAAGCAGAATAAAAGCGAAAAGAAATGAAAATGTTACACTACATT | 16910 | 0.16195799263651425 | No Hit |
| TCTTCACCCGCAGCTTCTGCTTCTCAGTCAGAAGGTTGTTGTCCTCATCC | 16760 | 0.16052134574736718 | No Hit |
| AGGCGGAGAGGGTCACTTCATCTTCTACTCCTCCCTTTATGGCATTGTTG | 16324 | 0.156345492122913 | No Hit |
| GTCCACGTCACACTTCATGATGGAGTTGAAGGTGGTCTCGTGGATGCCGC | 16321 | 0.15631675918513005 | No Hit |
| AACAAGCACAAAGCCAAGAAAACCAACGATGCCAGATACTGAATTTCTAT | 16219 | 0.15533983930051004 | No Hit |
| GGATCTGTCCTATAACAAGCTTAAAAACATACCAACTGTCAATGAAAACC | 16103 | 0.15422883237290297 | No Hit |
| GGCAATGAGCGGTTCCGGTGTCCGGAGGCGCTGTTCCAGCCTTCCTTCCT | 15883 | 0.15212175026882058 | No Hit |
| ACGTTTACCTGTCCATGTCTTACTACTTTGACCGCGATGATGTGGCTTTG | 15592 | 0.14933465530387524 | No Hit |
| CAGCATGTTCCCTCTCCTCATGAGATTGGTGAAGAAAGTATTTGGCAAAG | 15559 | 0.14901859298826287 | No Hit |
| TCAGGAGACGCTCGTAGCCCTCGCGCTTCTCCTCGGCCAATTCGCGGAAG | 15192 | 0.14550359693281636 | No Hit |
| TCAGGAGACGCTCGTAGCCCTCGCGCTTCTCCTCGGCCAGTTCGCGGAAG | 14999 | 0.14365511126878044 | No Hit |
| CTCCACCTACGGCGAGGGCGAGAGCGGCCCCATGGGCAACATCATGATCG | 14327 | 0.13721893320540152 | No Hit |
| GTCTCAACATCTGTGTTGGGGAGAGTGGAGACAGACTGACGCGAGCAGCC | 14217 | 0.13616539215336032 | No Hit |
| CGAGCCACAGCTCTGCCAGTACCCCAAGACTCAGCACTAGTCTGATGACC | 13609 | 0.13034218342935083 | No Hit |
| CGAAGCGGTTGTACTTGCGGATGTAGTGCAGATAGTCTCGGCGGATGACA | 13122 | 0.12567786986258664 | No Hit |
| CTCCGCCGCCTCCGCCGCAGACGCCGCCGCGATGCGCTACGTCGCCTCCT | 12952 | 0.12404967005488661 | No Hit |
| CGTGAATACCAAGACCTGCTCAATGTTAAGATGGCCCTTGACATTGAGAT | 12405 | 0.1188106977324636 | No Hit |
| CAGGGTGAAGGCCCACCCGTGGAGGCCAGACCCAAAGCCCACGGTACCGA | 12365 | 0.11842759189535772 | No Hit |
| CCACAACTTCCTTCTTCTCCTTTAAGTCCTTGGTGGTGATTTCGGAGCTG | 12013 | 0.1150562605288259 | No Hit |
| GGCATCAAAAGTGATGGACTGCCCATTGCTAGAGAAGACCTTCTCTCCTA | 11262 | 0.10786344843716283 | No Hit |
| CTGGAAGACGTACCTTTGTTGGAAGACATACCTTTGTTGGAAGACGTACC | 11260 | 0.10784429314530755 | No Hit |
| GGAGAAAAACAGCACCAAGCAGGAGATCCTGGCTGCTCTTGAGAAAGGCT | 11232 | 0.10757611905933342 | No Hit |
| CTGACAGTGTATCTAGCTTTGGAAAACACAGGGGTCTGCCCTGTGAGCTG | 11143 | 0.10672370857177282 | No Hit |
| CCAAGATCTTCTTGATGTCCTTGGCGCTGGGGGAGGAGTTGCCCCCTAGG | 11056 | 0.1058904533760675 | No Hit |
| TGCCATATCCACATGGAAAATCTCATCACCATCAAAGTCAAACATAAACT | 10838 | 0.10380252656384042 | No Hit |

## Adapter Content

Produced by FastQC (version 0.11.7)
